# A window of opportunity for abatacept in RA: is disease duration an independent predictor of low disease activity/remission in clinical practice?

**DOI:** 10.1007/s10067-017-3588-7

**Published:** 2017-03-01

**Authors:** Leslie R. Harrold, Heather J. Litman, Sean E. Connolly, Sheila Kelly, Winnie Hua, Evo Alemao, Lisa Rosenblatt, Sabrina Rebello, Joel M. Kremer

**Affiliations:** 1 University of Massachusetts Medical School, Worcester, MA USA; 2 Corrona, LLC, Southborough, MA USA; 3 Bristol-Myers Squibb, Princeton, NJ USA; 4 Albany Medical College and The Center for Rheumatology, Albany, NY USA

**Keywords:** Disease activity, Disease-modifying antirheumatic drugs (DMARDs), Non-TNFi biologic, Outcome measures, Registry, Rheumatoid arthritis

## Abstract

The objective of the study was to examine whether disease duration independently predicts treatment response among biologic-naïve patients with rheumatoid arthritis (RA) initiating abatacept in clinical practice. Using the Corrona RA registry (February 2006–January 2015), biologic-naïve patients with RA initiating abatacept with 12-month (±3 months) follow-up and assessment of disease activity (Clinical Disease Activity Index [CDAI]) at initiation and at 12 months were identified. The primary outcome was mean change in CDAI (ΔCDAI) from baseline to 12 months. Secondary outcomes at 12 months included achievement of low disease activity (LDA; CDAI ≤10 in patients with moderate/high disease activity at initiation) and remission (CDAI ≤2.8 in patients with low, moderate or high disease activity at initiation). Linear and logistic regression analyses were performed to examine the relationship between disease duration and response to abatacept. There were 281 biologic-naïve patients with RA initiating abatacept (disease duration 0–2 years, *n* = 107; 3–5 years, *n* = 45; 6–10 years, *n* = 50; >10 years, *n* = 79). Increased disease duration was associated with older age (*p* = 0.047), and the median number of prior conventional disease-modifying antirheumatic drugs used was lowest in the 0- to 2-year duration group (*p* < 0.001). Mean ΔCDAI (SE) ranged from −10.22 (1.19) for 0–2 years to −4.63 (1.38) for >10 years. In adjusted analyses, shorter disease duration was significantly associated with greater mean ΔCDAI (*p* = 0.015) and greater likelihood of achieving LDA (*p* = 0.048). In biologic-naïve patients with RA initiating abatacept, earlier disease (shorter disease duration) was associated with greater ΔCDAI and likelihood of achieving LDA.

## Introduction

Rheumatoid arthritis (RA) is a chronic, debilitating, progressive, inflammatory disease characterized by joint inflammation, which can lead to structural damage [1]. RA has a significant impact on patients’ abilities to perform activities of daily living [2], reduces physical and mental health-related quality of life, increases healthcare utilization and is associated with increased mortality [1]. The goal of RA therapy is to reduce disease activity, prevent long-term sequelae and improve functional status [2]. The treat-to-target paradigm is being widely advocated, specifically treatment acceleration in patients with active disease. The goal of this treatment paradigm is to achieve remission or, if remission is unattainable, low disease activity (LDA), based on validated outcome measures. Furthermore, it has been postulated that there is a ‘window of opportunity’ in early RA, during which treatment could alter the disease course before the inflammatory and autoimmune processes become established, irreversible damage has occurred and patients become increasingly refractory to treatment [3, 4]. Supporting this theory, patients with RA of longer disease duration generally do not respond as well to treatment with a biologic disease-modifying antirheumatic drug (bDMARD) as those with a shorter duration of disease [3]. Consequently, patients treated earlier have improved disease control and better outcomes compared with patients treated later in the disease course [4, 5].

Abatacept is a selective T-cell costimulation modulator available in intravenous and subcutaneous formulations and is indicated for the treatment of RA and juvenile idiopathic arthritis. The efficacy of abatacept has been demonstrated in patients naïve to methotrexate (MTX) or biologic therapy [5], as well as in treatment-experienced patients with an inadequate response to prior therapy with MTX [6] or tumour necrosis factor-α (TNF) inhibitors [7]. An exploratory post hoc analysis of pooled data from two abatacept trials in biologic-naïve patients with an inadequate response to MTX demonstrated that a higher percentage of patients with early RA (<2 years) achieved an American College of Rheumatology (ACR)70 response, Disease Activity Score in 28 joints (DAS28) (C-reactive protein) remission and clinically meaningful improvement in the Health Assessment Questionnaire (HAQ)-Disability Index versus patients with long-standing disease (>10 years) [8]. This relationship between disease duration and treatment response to abatacept in patients with RA has not been explored in patients cared for in routine clinical practice.

The objective of this analysis was to examine whether disease duration is an independent predictor of treatment response among biologic-naïve patients with RA initiating abatacept in a US national cohort.

## Patients and methods

### Patient population

The Corrona registry collects and analyses treatment and outcomes data for patients with chronic rheumatologic and dermatologic diseases. The Corrona RA registry is an independent, prospective, national, observational cohort study. Patients are recruited from 168 private and academic practice sites across 40 states in the USA, with 654 participating rheumatologists. As of 31 March 2016, the Corrona database included information on approximately 42,621 patients with RA. Data on 321,001 patient visits and approximately 141,984 patient-years of follow-up observation time have been collected. This study was carried out in accordance with the Declaration of Helsinki. All participating investigators were required to obtain full board approval for conducting non-interventional research involving human subjects with a limited dataset. Sponsor approval and continuing review was obtained through a central Institutional Review Board (IRB; New England Independent Review Board, NEIRB No. 120160610). For academic investigative sites that did not receive a waiver to use the central IRB, full board approval was obtained from the respective governing IRBs and documentation of approval was submitted to Corrona, LLC prior to initiating any study procedures. All registry patients were required to provide written informed consent and authorization prior to participating.

### Study population

This analysis included patients with RA >18 years of age who were biologic naïve and who had started treatment with abatacept during follow-up in the Corrona RA registry between February 2006 and January 2015. Eligible patients had to have a follow-up visit at 12 months (±3 months) after the initiation of treatment (using the visit closest to 12 months if there was >1 visit) and Clinical Disease Activity Index (CDAI) measured at or prior to the initiation of treatment. If the physician reported a treatment start date, CDAI score was measured within 4 months prior to initiation. If the physician did not report a start date for treatment, disease activity from the prior visit was used if this visit was within 6 months of the date of initiation. Other inclusion criteria were measurement of CDAI score at 12 months (±3 months) or at the time of switch if this occurred prior to the 12-month visit and no prior bDMARD use.

### Assessments and data collection

Patient demographics, baseline characteristics and clinical outcomes were assessed across four disease duration groups (0–2, 3–5, 6–10, >10 years). Disease duration was collected from the rheumatologist based on the year of RA onset and the visit of initiation. Data were collected from patients and their treating rheumatologists using standard clinical research forms at the time of the clinical encounter. These forms gather information on disease severity and activity (including components of ACR response criteria), medical comorbidities, use of medications including conventional (c)DMARDs and adverse events. Data elements collected in the registry that were relevant to this study included components of CDAI (swollen joint count, 28 tender joint count, physician global assessment and patient global assessment), patient assessment of pain, DAS28 (erythrocyte sedimentation rate; ESR) and the modified (m)HAQ assessing physical function [9]. Data on demographics, insurance status, comorbid conditions, RA disease characteristics and RA medications were available for >98% of patients. The scope of the data collected and comparison with other registries have been previously described [10].

### Study outcomes

The primary outcome was mean change in CDAI (ΔCDAI) at 12 months (CDAI score at 12 months minus CDAI score at initiation). The secondary outcomes were achievement of LDA (CDAI score ≤10) among patients who initiated abatacept with moderate or high disease activity and achievement of remission (CDAI score ≤2.8) in those who initiated abatacept with low, moderate or high disease activity. Discontinuations and switching status were also examined. Patients were categorized into one of three mutually exclusive groups: those who switched from abatacept to a new biologic, those who discontinued abatacept without initiating a new biologic before their 12-month visit and those who remained on abatacept until their 12-month visit.

### Statistical analysis

Baseline characteristics of patients in the different disease duration groups were compared using descriptive statistics. A two-sided 5% significance level was used to assess statistical differences. For patients who switched agents before 12 months, the last observation before the switch was used to calculate mean ΔCDAI. Switchers were imputed as non-responders for calculation of LDA and remission. For patients who discontinued an agent and did not start another before 12 months and those who continued on their original agent through their 12-month visit, measurement at the 12-month visit was used to calculate outcomes. Mean ΔCDAI from baseline was calculated by disease duration group. The rates of LDA/remission and remission at 12 months by disease duration group were estimated (unadjusted). *p* values were based on omnibus tests of any differences in the duration groups. Fisher’s exact test was used when there were small counts, as for switching status. Multivariable linear regression models were fit to assess the association of ΔCDAI from baseline by disease duration group. Multivariable logistic regression models were conducted to assess the association of disease duration and LDA/remission and remission at 12 months. Baseline characteristics that were indicative of a difference (*p* < 0.1) across the disease duration groups, as well as characteristics that were chosen a priori by investigators (age, sex, baseline CDAI score and number of prior cDMARDs), were used as covariates in the models. Of note, MTX use did not meet criteria for inclusion in the adjusted models. However, as MTX use may influence results, we re-ran the above-mentioned adjusted models with the addition of MTX. The results were unchanged (data not shown) from the original models and thus are not included in this manuscript.

## Results

### Patient disposition and baseline characteristics

There were a total of 281 abatacept initiators who were biologic naïve and met the inclusion criteria (disease duration 0–2 years, 107 [38.1%]; 3–5 years, 45 [16.0%]; 6–10 years, 50 [17.8%]; >10 years, 79 [28.1%]). In general, baseline characteristics were similar across the four disease duration groups (Table 1), with a similar percentage of female patients (76–89%) and similar distributions for level of education, comorbid conditions, smoking status and morning stiffness. The average weight, body mass index, blood pressure and most of the disease activity measures including CDAI, DAS28 (ESR), patient pain, mHAQ and patient-reported fatigue were also similar across the disease duration groups. Patients with disease duration >10 years were older (median [IQR] 67 [16] years), were on average younger at onset of RA (mean [SD] 46.5 [13.2] years) and had higher prior cDMARD use (median [IQR] of 2 [2]) versus patients in other disease duration groups.

**Table 1:** Patient demographics and clinical characteristics by disease duration group in biologic-naïve abatacept initiators

Characteristic	0–2 years, *n* = 107	3–5 years, *n* = 45	6–10 years, *n* = 50	>10 years, *n* = 79	*p* ^a^
Female sex, *n* (%)	88 (82.2)	34 (75.6)	39 (78.0)	70 (88.6)	0.24
Age, years, median (IQR)	61.0 (21)	65.0 (18)	64.5 (15)	67.0 (16)	0.047
Duration of RA, median (IQR)	1.0 (1)	4.0 (1)	8.0 (3)	19.0 (14)	<0.001
Age at onset, years, mean ± SD	59.0 ± 14.0	57.6 ± 13.6	55.8 ± 11.6	46.5 ± 13.2	<0.001
Race, *n* (%)					0.018
White	91 (85.0)	29 (64.4)	40 (80.0)	62 (80.5)^b^	
Hispanic	8 (7.5)	10 (22.2)	5 (10.0)	12 (15.6)^b^	
African-American	5 (4.7)	6 (13.3)	1 (2.0)	3 (3.9)^b^	
Asian	2 (1.9)	0	2 (4.0)	0	
Other	1 (0.9)	0	2 (4.0)	0	
Smoking status, *n* (%)^c^					0.23
Never	52 (50.0)	25 (55.6)	22 (44.0)	45 (57.0)	
Previous	40 (38.5)	12 (26.7)	18 (36.0)	29 (36.7)	
Current	12 (11.5)	8 (17.8)	10 (20.0)	5 (6.3)	
Weight, lbs., median (IQR)	164 (53)	166 (45)	174 (48)	163 (49)	0.35
Work status, *n* (%)					0.002
Full time	43 (40.2)	10 (22.2)	12 (24.0)	16 (20.3)	
Part time	15 (14.0)	9 (20.0)	4 (8.0)	5 (6.3)	
Not working outside home	13 (12.1)	6 (13.3)	5 (10.0)	3 (3.8)	
Student	1 (0.9)	1 (2.2)	0	2 (2.5)	
Disabled	4 (3.7)	6 (13.3)	8 (16.0)	12 (15.2)	
Retired	31 (29.0)	13 (28.9)	21 (42.0)	41 (51.9)	
Number of prior cDMARDs (including current cDMARD), median (IQR)	1 (1)	2 (1)	2 (1)	2 (2)	<0.001
Presently on MTX, *n* (%)	78 (72.9)	30 (66.7)	28 (56.0)	56 (70.9)	0.19
Comorbid conditions, *n* (%)					
Hypertension	38 (35.5)	17 (37.8)	19 (38.0)	38 (48.1)	0.36
Diabetes	9 (8.4)	4 (8.9)	5 (10.0)	4 (5.1)	0.73
Malignancy^d^	14 (13.1)	2 (4.4)	7 (14.0)	11 (13.9)	0.39
CV disease	5 (4.7)	2 (4.4)	3 (6.0)	8 (10.1)	0.45
Serological status					
CCP positive, *n* (%)^e^	25 (55.6)	8 (66.7)	11 (73.3)	14 (73.7)	0.43
RF positive, *n* (%)^f^	35 (71.4)	18 (85.7)	17 (63.0)	37 (74.0)	0.37
CDAI score, median (IQR)^g^	18 (16.5)	12.5 (19.5)	12.5 (17)	13 (16.9)	0.13
Patient pain (0–100), median (IQR)^h^	40 (45)	30 (40)	35 (35)	32.5 (50)	0.70
DAS28 (ESR), median (IQR)^i^	4 (1.9)	3.6 (3.3)	4.3 (1.5)	3.9 (2.1)	0.90
Patient-reported fatigue (0–100), median (IQR)^j^	40 (55)	35 (55)	55 (50)	40 (55)	0.92
mHAQ, median (IQR)^k^	0.25 (0.69)	0.13 (0.63)	0.38 (0.50)	0.25 (0.63)	0.55
Morning stiffness, *n* (%)					0.30
None	25 (23.4)	10 (22.2)	10 (20.0)	25 (31.6)	
<1 h	29 (27.1)	17 (37.8)	12 (24.0)	25 (31.6)	
≥1 h	53 (49.5)	18 (40.0)	28 (56.0)	29 (36.7)	

*CDAI* Clinical Disease Activity Index, *cDMARD* conventional disease-modifying antirheumatic drug, *CV* cardiovascular, *DAS28* Disease Activity Score in 28 joints, *ESR* erythrocyte sedimentation rate, *IQR* interquartile range, *mHAQ* modified Health Assessment Questionnaire, *MTX* methotrexate, *RA* rheumatoid arthritis, *SD* standard deviation

^a^ *p* values were from Wilcoxon rank sum tests in the case of comparing medians across the duration groups. When only means are reported, analysis of variance was used to compare means across the duration groups

^b^ *n* = 77

^c^ *n* = 104, *n* = 45, *n* = 50 and *n* = 79, respectively

^d^History of malignancy includes history of lung cancer, breast cancer, skin cancer (includes melanoma, basal and squamous cell skin cancer), lymphoma or other cancer

^e^ *n* = 45, *n* = 12, *n* = 15 and *n* = 19, respectively

^f^ *n* = 49, *n* = 21, *n* = 27 and *n* = 50, respectively

^g^ *n =* 107, *n* = 45, *n* = 49 and *n* = 79, respectively

^h^ *n* = 107, *n* = 45, *n* = 50 and *n* = 78, respectively

^i^ *n* = 60, *n* = 18, *n* = 22 and *n* = 30, respectively

^j^ *n* = 77, *n* = 27, *n* = 26 and *n* = 46, respectively

^k^ *n* = 104, *n* = 43, *n* = 50 and *n* = 79, respectively

### Efficacy

The results for the primary and secondary efficacy outcomes are presented in Table 2. In the unadjusted analysis, the greatest ΔCDAI from baseline occurred in the group of patients with the shortest disease duration (0–2 years: mean ΔCDAI [SE]: −10.22 [1.19]) and the smallest change occurred in the group with the longest disease duration (>10 years; −4.63 [1.38]; overall *p* = 0.017). Response rates for the binary outcomes of LDA and remission did not differ significantly according to disease duration group (*p* = 0.21 and 0.23, respectively; Table 2).

**Table 2:** Primary and secondary efficacy outcomes

Outcomes	0–2 years, *n* = 107	3–5 years, *n* = 45	6–10 years, *n* = 50	>10 years, *n* = 79	*p*
Unadjusted outcomes					
Mean ΔCDAI from baseline (95% CI)	−10.22 (−12.55, −7.89)	−7.82 (−11.41, −4.24)	−6.16 (−9.56, −2.76)	−4.63 (−7.34, −1.92)	0.017
Achievement of LDA,^a^ *n*/*N* (%)	53/91 (58.2)	17/32 (53.1)	15/33 (45.5)	23/56 (41.1)	0.21
Achievement of remission,^b^ *n*/*N* (%)	29/103 (28.2)	11/40 (27.5)	12/48 (25.0)	11/72 (15.3)	0.23
Adjusted outcomes^c^					
Difference in mean ΔCDAI from baseline (95% CI)	5.07 (1.88, 8.26)	4.03 (0.19, 7.86)	2.19 (−1.45, 5.83)	Reference	0.015
Achievement of LDA,^a^ OR (95% CI)	2.76 (1.28, 5.97)	2.44 (0.92, 6.51)	1.36 (0.53, 3.49)	Reference	0.048
Achievement of remission,^b^ OR (95% CI)	3.12 (1.30, 7.46)	2.65 (0.97, 7.28)	1.76 (0.67, 4.62)	Reference	0.055

*ΔCDAI* change in CDAI, *cDMARD* conventional disease-modifying antirheumatic drug, *CDAI* Clinical Disease Activity Index, *CI* confidence interval, *LDA* low disease activity, *OR* odds ratio

^a^CDAI ≤10 among those with moderate or high disease activity

^b^CDAI ≤2.8 among those with low, moderate or high disease activity

^c^Adjusted for age, sex, baseline CDAI and number of prior cDMARDs used

After adjustment for age, sex, baseline CDAI score and prior number of cDMARDs used, the significant difference between the disease duration groups in ΔCDAI from baseline remained (0–2 years, mean ΔCDAI [SE], −9.65 [1.02]); >10 years, −4.58 [1.19]; overall *p* = 0.015). In addition, there was a significant difference in achievement of LDA across the four groups, with the group of patients with disease duration of 0–2 years being nearly three times as likely to reach LDA at 12 months following abatacept initiation as the group with >10 years of disease duration (odds ratio [95% confidence interval] 2.76 [1.28, 5.97]; *p* = 0.048). Similarly, those with 0–2 years of disease duration were three times more likely to achieve remission at 12 months than those with >10 years of disease duration (odds ratio [95% confidence interval] 3.12 [1.30, 7.46]; *p* = 0.055). Of note, the proportion of patients who remained on abatacept at 12 months was similar across the disease duration groups, ranging from 67 to 74% (*p* = 0.60; Table 3).

**Table 3:** Abatacept status at 12 months

	0–2 years, *n* = 107	3–5 years, *n* = 45	6–10 years, *n* = 50	>10 years, *n* = 79	*p* ^a^
Switching status					0.60
Remained on abatacept at 12 months	79 (73.8)	30 (66.7)	35 (70.0)	57 (72.2)	
Discontinued abatacept^b^	13 (12.1)	6 (13.3)	10 (20.0)	14 (17.7)	
Switched from abatacept^c^	15 (14.0)	9 (20.0)	5 (10.0)	8 (10.1)	

Values are *n* (%)

^a^Fisher’s exact test

^b^Discontinuation of abatacept without a new biologic started

^c^Discontinuation with a new biologic started

## Discussion

In biologic-naïve patients with RA, unadjusted modelling revealed a significant difference in ΔCDAI from baseline between patients with shorter versus longer disease duration. However, unadjusted response rates for LDA and remission were similar between disease duration groups. ΔCDAI from baseline remained significant in the multivariable model adjusted for age, sex, baseline CDAI and number of prior cDMARDs. Furthermore, the relationship between disease duration and achievement of LDA was statistically significant, while remission was borderline significant in the adjusted models.

Disease duration has been identified as a predictor of treatment response in patients with RA in both this manuscript and other publications [3], highlighting the importance of early treatment with both cDMARDs and bDMARDs to achieve remission or LDA when remission is not attainable [11]. Our results are similar to those from other studies that demonstrated that earlier treatment with non-bDMARDs or TNF inhibitors was associated with a greater likelihood of remission [12, 13]. Some interventional trials reported that earlier treatment with abatacept is associated with better outcomes [8, 14], although one post hoc analysis found no association between disease duration and response [15]. The difference in these findings may reflect variations between trials in disease duration (e.g. patients with early RA versus patients with established RA) and treatment history (e.g. biologic naïve versus biologic experienced, MTX responders versus those with an inadequate MTX response and TNF-inhibitor responders versus non-responders). These results, showing a greater benefit in patients with shorter disease duration at treatment initiation, are consistent with the proposed early window of opportunity during which there is potential to alter the disease course in early RA, which otherwise becomes diminished as the disease becomes established [1].

Earlier treatment is generally accepted to be associated with improved long-term outcomes. Communication with patients about the long-term goals of therapy is critical given the apparent window of opportunity for altering the disease course. Therefore, physicians need to educate patients on the treat-to-target paradigm, the ACR treatment guidelines and the benefits of escalating therapy until remission or LDA is achieved, particularly early in the disease course as this can improve long-term outcomes [2]. Registry studies provide valuable data on treatment outcomes in a large cohort of patients in the real-world setting. This analysis of patients in a US registry provides further long-term (exceeding 10 years in some patients) evidence to support early initiation of a biologic therapy, in this case, abatacept.

A strength of this study is that it is the largest national US registry in RA that contains both patient- and provider-reported measures. As with any observational study, there are limitations. For example, patients were not randomly assigned to the disease duration groups, so there could have been unmeasured differences between the cohorts. To attempt to address this, we have focused on a homogeneous group of biologic-naïve patients only.

In conclusion, these results show that, in a typical US clinical practice setting, abatacept was effective in biologic-naïve patients with RA who had a range of disease durations at treatment initiation. However, the magnitude of the response to abatacept, including reduction in disease activity and achievement of LDA/remission, was greater in patients with shorter versus longer disease duration.
